# Nearly full-length genome sequence of a novel astrovirus isolated from chickens with ‘white chicks’ condition

**DOI:** 10.1007/s00705-016-2940-6

**Published:** 2016-06-23

**Authors:** Joanna Sajewicz-Krukowska, Katarzyna Domanska-Blicharz

**Affiliations:** Department of Poultry Diseases, National Veterinary Research Institute, Al. Partyzantow 57, 24-100 Pulawy, Poland

**Keywords:** Astrovirus, Chicken, Phylogenetic analysis, Recombination

## Abstract

Avian astroviruses (aAstVs) are divided into three species, *Avastrovirus 1*, *Avastrovirus 2*, *and Avastrovirus 3*, but there are a few strains are waiting to be assigned to an official taxonomic group. This study presents the molecular characterization of chicken astrovirus (CAstV), PL/G059/2014, which is involved in the induction of “white chicks” condition. The 7382-nucleotide-long genome sequence was determined by next-generation sequencing using an Illumina MiSeq System. Phylogenetic analysis showed that it has the characteristics that are typical of avian astroviruses. However, overall degree of nucleotide sequence identity was 43.6 % to 73.7 % between PL/G059/2014 and other available genome sequences of aAstV strains. The amino acid sequences of the proteins encoded by ORF1a and ORF1b of the studied strain were very similar (86.5-93.8 % identity) to those of CAstVs 4175 and GA2011, but they were only 32.7-35.2 % identical in the case of ORF2, which is used officially for astrovirus species demarcation. These features could suggest that the PL/G059/2014 strain should be assigned to a new species in the genus *Avastrovirus*. Moreover, the different phylogenetic topology of PL/G059/2014 and its nucleotide sequence similarity in different genomic regions could suggest that a recombination event occurred during its evolution and that it has ancestors in common with duck astroviruses.

Astroviruses are small viruses with a diameter of approximately 28–30 nm. Their single-stranded RNA genome ranges from 6.8 to 7.9 kb and contains three open reading frames (ORFs) with a 5′ untranslated region (UTR) and a 3′ UTR, and a poly-A tail. ORF1a encodes the non-structural (NS) polyprotein, while ORF1b and ORF2 encode the RNA-dependent RNA polymerase (RdRp) and the viral capsid structural protein, respectively [4, 7]. These viruses belong to the family *Astroviridae*, which is divided into two genera: *Mamastrovirus* and *Avastrovirus*, depending on the organisms they infect. For many years, this criterion was also used for further classification of astrovirus strains into separate species. Within the genus *Avastrovirus*, such division distinguished two turkey astroviruses, turkey astrovirus type 1 (TAstV-1) and type 2 (TAstV-2); two chicken viruses, avian nephritis virus (ANV) and chicken astrovirus (CAstV); and duck astroviruses (DAstV) [15]. However, a number of astroviruses have been found recently in different bird species; moreover, it turned out that they could be transmitted between species [6]. The new official astrovirus classification system is based on the amino acid sequence of viral capsid protein and most of the above-mentioned astroviruses have been assigned to three species: *Avastrovirus 1*, *Avastrovirus 2, and Avastrovirus 3*. When this classification was created, due to the lack of available sequences, CAstVs were referred to as “related viruses which may be members of the *Avastrovirus* genus but have not been approved as species” [8]. In the meantime, the full genomes of two CAstV strains and the capsid gene of additional CAstVs from different geographical locations have been sequenced [3, 9, 18]. A phylogenetic analysis of available CAstV ORF2 sequences showed that they clustered in a group provisionally representing a new species [9]. This avastrovirus group also varies because different subgroups can be distinguished [3, 18]. In recent years, there have been more viruses awaiting formal classification, such as duck hepatitis virus type 2 (DHV-3), DHV-3-like astroviruses, duck astrovirus CPH (DAstV CPH), duck astrovirus SL-like, duck astrovirus YP-like and astroviruses detected in wild birds [5, 10–12, 22].

This report presents the genetic characterization of a novel astrovirus recently detected in Poland. This CAstV strain was associated with increased mortality of embryos and chicks, as well as weakness and white plumage of hatched chicks, a disease described as ‘white chicks’ condition [16]. The virus was propagated (isolated) on embryonated specific-pathogen-free (SPF) chicken eggs and then used in experimental reproduction of this condition in SPF layer chickens [14].

We used real-time RT-PCR to identify the viral agent according to previous protocols [17]. Six 10-day-old SPF chicken embryos (VALO BioMEDIA, Germany) were inoculated (0.2 ml/egg) with 10 % suspension of organ sample homogenate in which CAstV was detected. The inoculated eggs were incubated at 37 °C and candled daily for 5 days. After cooling, allantoic fluids and altered organs of embryos were harvested. Applied molecular methods revealed a large amount of CAstV genome (Ct value of about 15-16) in these samples. The isolated virus was designated as PL/G059/2014. The allantoic fluid was then used in the experimental reproduction of ‘white chicks’ condition and for extraction of RNA for further molecular characterization. We sequenced a nearly full-length genome of the viral isolate PL/G059/2014 at the commercial service Genomed Sp. z o.o. (Poland). Library preparation, further investigation and “next-generation sequencing” using an Illumina MiSeq System (Illumina Inc., San Diego, USA) were performed. Reads were assembled into contigs and compared to sequences in the GenBank nucleotide and protein databases using BLASTn/BLASTx. The CLC Genomics Workbench v7.0 was used for all downstream bioinformatic analyses.

The sequence of the nearly full-length genome of the CAstV PL/G059/2014 strain consisted of 7382 nt, excluding the poly(A) tail, and was 301 nt shorter than the full sequence of the reference CAstV GA2011 strain (GenBank accession no. JF414802). The nucleotide composition of the nearly full-length sequence of the CAstV strain is 31 % A, 12 % G, 45 % T and 12 % C. The G/C content is 24 %. NCBI Sequin was used for ORF prediction and genome annotation (Fig. 1).

**Fig. 1:**
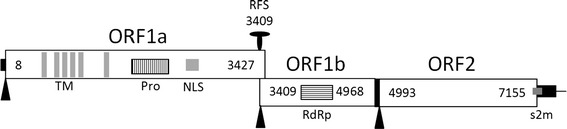
Predicted genome organization of CAstV PL/G059/2014. Three ORFs with their location and the motifs typical for astroviruses are shown. The translation start sites of ORFs are indicated by black triangles. Black bars are the untranslated regions and the 24-nt spacer between the stop and start codons of ORF1b and ORF2, respectively. ORF, open reading frame; TM, transmembrane domain; Pro, protease; NLS, nuclear localization signal; RdRp, RNA-dependent RNA polymerase; RFS, ribosomal frameshift signal; s2m, stem-loop-II-motif

The PL/G059/2014 had a typical AstV genome structure with three sequential, overlapping ORFs: ORF1a (8–3427, corresponding to positions 22–3441 of the reference GA2011 sequence), OFR1b (3409-4968, positions 3423–4982 of the reference GA2011 sequence) and ORF2 (4993-7155, positions 5007–7169 of the GA2011 sequence). The nearly complete genome sequence contained a partial 5′ untranslated region (UTR) (nt 1-7, positions 15–21 of the reference GA2011 sequence), and 3′UTR (nt 7156-7382, positions 7315-7521 of the reference GA2011 sequence). Examination of the first nucleotides of the virus revealed the presence of two in-frame AUG codons of ORF1a, beginning at position 8 and 20, respectively (GCGATGGCCCAGGCCATGG8-23), both with the strongest positive effects on translation. ORF1a is 3427 nt long and encodes a polypeptide of 1139 amino acids (aa) with a calculated M_r_ of 129.77 kDa. As in the case with other astroviruses, there is an overlap region between ORF1a and ORF1b (nt 3409 to 3427), which contains the heptameric frameshift sequence AAAAAAC (nt 3418-3424) known as RFS (ribosomal frameshift signal). ORF1b is 1559 nt long and encodes a polypeptide of 519 aa with a calculated M_r_ of 60.41 kDa. As is typical for astroviruses, a 24-nt spacer between the stop codon of ORF1b and the start codon of ORF2 with a highly conserved CCGAA pentamer at positions 4980-4984 is also present. ORF2 is 2162 nt long and encodes a capsid protein precursor of 720 aa with a calculated M_r_ of 80.01 kDa. Rfam analysis of (http://rfam.xfam.org) revealed the presence of a highly conserved coronavirus 3′ stem-loop-II-like motif (s2m) consisting of 23 nt of ORF and the adjacent 18 nt of the 3′UTR (7136-7178). The exact role of s2m remains obscure, but recently, it was described as genetic element that, through an RNA-interference-like mechanism, influences gene expression in the infected organism, providing some kind of selective advantage for the virus [21].

The predicted nonstructural proteins of PL/G059/2014 contained the characteristic aa motifs that are conserved in other astroviruses: a serine protease (Pro) motif at position 691 (GNSG), a nuclear localization signal (NLS) motif at position 802 (KKKGKTK), and four RdRp motifs at positions 266 (DWTRFD), 328 (GNPSG), 378 (YGDD), 406 (FGMWVK). Six possible transmembrane domains (TM) were detected in the ORF1a protein at the following positions: 220-240, 375-395, 414-436, 444-462, 477-495, and 660-686.

A phylogenetic analysis of the nearly complete nucleotide sequence was conducted to investigate the relationship of CAstV PL/G059/2014 to other astroviruses. The amino acid sequences of all three ORFs were also compared phylogenetically. All analyses were performed using MEGA version 6.06 [20]. The ClustalW method was used for nucleotide and deduced amino acid sequences alignments, and the neighbour-joining method with 1000 bootstrap replicates was used for generation of phylogenetic trees. Nucleotide and aa sequences of available AstV genomes, representative of putative avian and non-avian astrovirus species, were obtained from GenBank and included in the analysis.

Analysis of the nearly complete nucleotide sequence revealed that PL/G059/2014 was in the same branch of the phylogenetic tree as the only two available CAstVs strains, GA2011 and 4175 (Fig. 2a). The same alignments were obtained in ORF1a and ORF1b phylogenetic trees, with PL/G059/2014 most closely related to the CAstVs GA2011 and 4175 (Fig. 2b and c). With regard to ORF2, previous analysis of available CAstVs showed the existence of two chicken astrovirus groups, namely A and B [3, 18]. The above-mentioned CAstVs GA2011 and 4175, along with other ones from the UK and India, formed group B. However, the PL/G059/2014 strain was found to be in group A with CAstVs/P22-18.8.00 and VF08-36 (Fig. 2d). It is unknown whether the other strains of group A in the analysis of individual ORFs clustered similarly to the Polish strain, since there are only ORF2s available in the public domain. Genome sequence comparison confirmed the results obtained in phylogenetic analysis (Table 1).

**Fig. 2:**
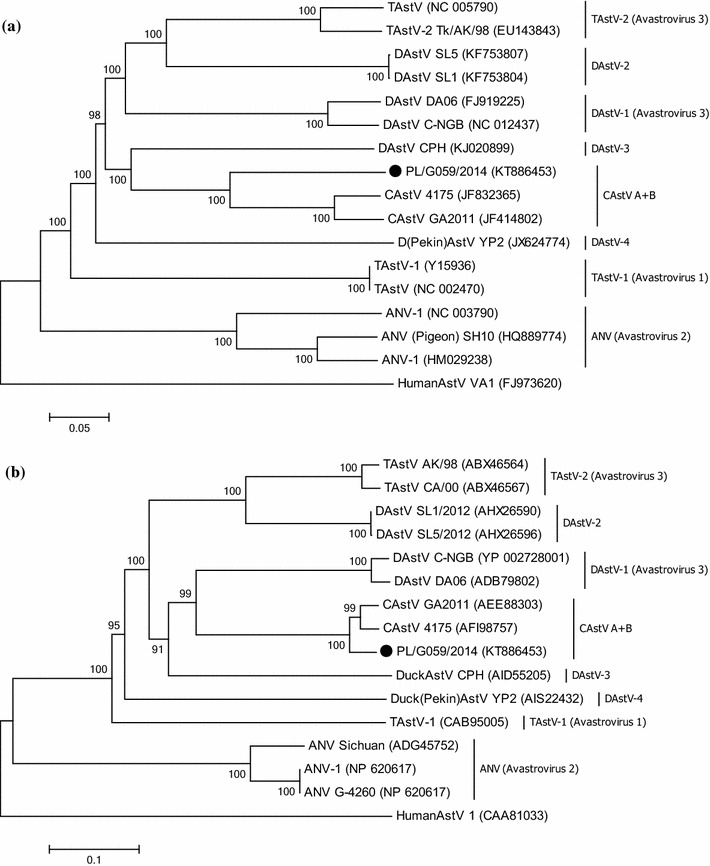

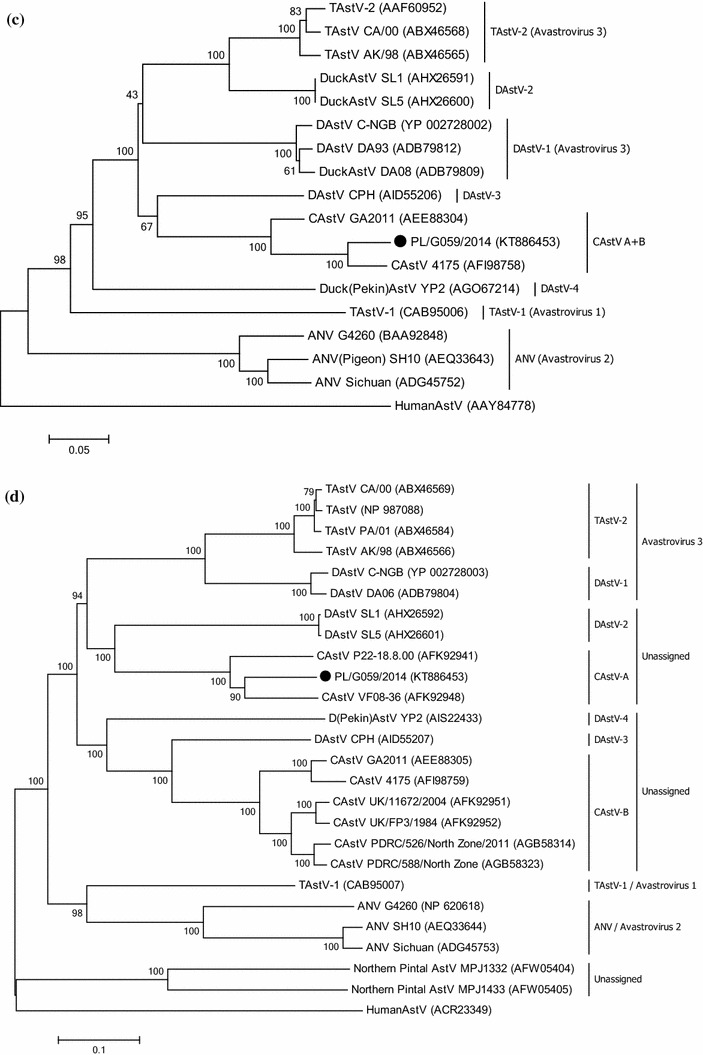
Phylogenetic relationships between the CAstV PL/G059/2014 isolate from this study and other astroviruses. The analysis was based on the nucleotide sequences of the nearly complete genome (a) and on amino acid sequences of the complete ORF1a (b), ORF1b (c) and ORF2 (d) regions. Human astrovirus (HumanAstV-1) was used as an outgroup. The trees were constructed using MEGA 6, using the neighbor-joining method and 1000 bootstrap replicates (bootstrap values shown on the tree). GenBank accession numbers of the sequences are indicated in parentheses. The PL/G059/2014 isolate determined in this study is indicted by a black dot

**Table 1:** Evolutionary divergence between sequences

Virus	GenBank accession number	Percent identity to PL/G059/2014			
		Genome (nt)	ORF1a (aa)	ORF1b (aa)	ORF2 (aa)
CAstV 4175	JF832365	73.7	93.8	93.2	32.7
CAstV GA2011	JF414802	71.7	93.8	86.5	35.2
TAstV-2	EU143843	52.8	47.4	61.8	44.3
TAstV-1	Y15936	49.9	40.2	51.7	40.2
ANV-1	NC003790	43.7	26.6	49.0	27.8
DAstV CPH	KJ020899	56.7	53.3	68.1	41.9
DAstV SL5	KF753807	53.9	48.0	65.1	49.4
DAstV C-NGB	NC012437	55.8	58.6	62.3	43.8
DAstV YP2	JX624774	50.2	43.1	56.4	41.2
BatAstV	EU847155	39.8	12.8	37.1	18.1
HumanAstV VA1	FJ973620	37.0	13.9	34.7	19.3
OvineAstV	NC002469	36.3	15.0	34.9	18.9
MinkAstV	GU985458	36.3	15.8	34.4	18.9
P22-18.8.00	JN582318	n/a	n/a	n/a	80.8
VF08-36	JN582325	n/a	n/a	n/a	82.1
Northern pintal AstV (MPJ1433 isolate)	JX985651	n/a	n/a	n/a	20.3

The nearly full-length nucleotide genome sequence of PL/G059/2014 has the closest similarity to those of CAstVs GA2011 and 4175, at the level of 71.7-73.7 %. The next astrovirus strains with similarity to PL/G059/2014 were DAstVs CPH and C-NGB, with nucleotide sequence identity of 56.7 % and 55.8 %, respectively (Table 1). The amino acid sequences of ORF1a and ORF1b of PL/G059/2014 shared the highest identities of 86.5 % to 93.8 % with the published sequences of CAstVs GA2011 and 4175 as well (Table 1). However, the aa sequence similarity of ORF2 to these chicken astrovirus strains was rather low, at the level of 32.7-35.2 %. The PL/G059/2014 strain had the highest ORF2 aa sequence identity to P22-18.8.00 and VF08-36 strains, and it ranged between 80.8 % and 82.1 %. The next astrovirus strains with similarity to PL/G059/2014 were DAstV-2 strains (representative SL5 in Table 1), with 49.4 % nucleotide sequence identity. In turn, the studied strains showed the lowest similarity, at the level of 20.3 %, to astroviruses detected in wild aquatic birds in Cambodia and Hong Kong [5].

The different topology of the PL/G059/2014 strain in the phylogenetic trees of ORF1a/ORF1b and ORF2 as well as nt similarity in different genomic regions to different astrovirus strains suggested that recombination among the astroviruses might have occurred in the field. The ORF1a of PL/G059/2014 was most closely related to that of DAstV-1; and ORF1b, to that of DAstV-3. This situation was also observed in ORF1a and ORF1b of other CAstV strains, GA2011 and 4175. However, in the case of these two CastV strains, similarity of ORF2 to that of DastV-3 was also observed. Surprisingly, the ORF2 of PL/G059/2014 was more closely related to a duck astroviruses, DastV-2. This may suggest that the nonstructural and structural protein genes of the PL/G059/2014 came from three different ancestor astroviruses, all hosted by ducks. A recombination event between astroviruses with different hosts of origin as one of the main mechanisms of virus evolution was previously suggested [11, 13, 19, 23]. Taking into consideration that many different avastroviruses have recently been detected in ducks and that their genomes showed phylogenetic relationships to poultry astroviruses, it seems probable that ducks may play an important role in the epidemiology of astrovirus, similar to the case with avian influenza virus [1].

The mean amino acid genetic distances (p-dist) based on the analysis of the aa sequence of ORF2 with strains belonging to three official avastrovirus species are as follows: 0.576-0.583 with the Tk/AK/98 and C-NGB strains of *Avastrovirus 3*, 0.600 with TAstV-1 of *Avastrovirus 1*, and 0.725 with G4260 of *Avastrovirus 2* (Table 2). On the other hand, the p-dist values with chicken astroviruses GA2011 and 4175 were 0.613-0.644, but only 0.178-0.192 with CAstVs P22-18.8.00 and VF08-36 (Table 2). These p-dist values are even lower than those proposed by the International Committee on Taxonomy of Viruses as criteria for avastrovirus species demarcation (p-dist of 0.576-0.742, and 0.204-0.284 between and within avastrovirus groups), so it seems that strains PL/G059/2014, Dutch P22-18.8.00 and British VF08-36 belong to the same avastrovirus species but are separate from CAstV GA2011 and 4175 [8].

**Table 2:** Mean amino acid genetic distances (p-dist) based on the analysis of aa sequences of ORF2 of the PL/G059/2014 strain and avian AstV strains of the official species/provisional groups

Official species/provisional group	Virus strain	p-dist
*Avastrovirus 3*	Tk/AK/98	0.576
*Avastrovirus 1*	DAstV C-NGB	0.583
	TAstV-1	0.600
*Avastrovirus 2*	G4260	0.725
CAstV B	CAstV 4175	0.644
	CAstV GA2011	0.613
CAstV A	P22-18.8.00	0.192
	VF08-36	0.178
DAstV 3	DAstV CPH	0.581
DAstV 4	DAstV YP2	0.587
DAstV 2	DAstV SL5	0.506
DAstV 1	DAstV C-NGB	0.583
Wild birds	Northern Pintal AstV (MPJ1433 isolate)	0.797

PL/G059/2014 has been detected in chickens in which the only symptom detected was an impairment in feather pigmentation – the disease known as ‘white chicks’ condition. With regard to ORF2 protein structure, the most closely phylogenetically related strains were European CAstV strains that are responsible for enteric and respiratory problems in chickens, as well as hatchability issues [2, 18, 22]. We detected some differences in the capsid protein sequences of these strains, but their implications for pathogenicity are unknown. To investigate this question, whole astrovirus genome sequences should be compared, but they are currently unavailable. However, it should be remembered that pathogenicity could be influenced by the dose and route of inoculation, age and breed of chickens, level of maternally derived or acquired antibodies, and co-infection with other pathogens. For these reasons, conclusions about the pathogenicity of the virus based on their genome sequences should be made with caution.

In conclusion, the present work describes the nearly full-length genome of chicken astroviruses responsible for ‘white chicks’ condition recently identified in Poland. Based on the criteria for species demarcation recommended recently by the ICTV, the virus should be classified as a member of a new species within the genus *Avastrovirus*. Its genomic similarity to different astroviruses supports the previous suggestion that recombination events have played a role in the evolution of astroviruses. The data presented here also suggest a need for redefinition of taxonomic classification criteria for avastroviruses.
